# From one-size-fits-all to context-specific: the 10-day observation rule-a need for change

**DOI:** 10.3389/fpubh.2026.1934845

**Published:** 2026-09-03

**Authors:** Xin Wang

**Affiliations:** Wuxi No.8 People’s Hospital, Wuxi, China

**Keywords:** 10-day observation rule, guideline-associated rabies phobia, implementation gap, post-exposure prophylaxis, rabies

## Abstract

WHO’s ‘Zero by 30’ strategic plan relies on post-exposure prophylaxis guidelines designed for high-resource settings. This policy brief argues that the implementation gap of the 10-day observation rule in high-burden countries stems from fundamental infeasibility, exposing two paradigm defects in WHO guideline development: one-size-fits-all thinking and insufficient attention to implementation science. When the public misinterprets the 10-day rule as “wait and see” rather than “initiate PEP immediately while observing the animal,” three biological facts render this misinterpretation fatal: symptomatic rabies is nearly 100% fatal and untreatable; a single vaccine dose cannot promptly induce sufficient neutralizing antibodies; and the incubation period for craniofacial Category III bites can be as short as 5–10 days—shorter than the full observation window. Waiting for animal observation without initiating PEP during this window means missing the only opportunity for effective prevention. Evidence from multiple countries shows that the rule’s preconditions are almost never met in settings where 95% of rabies deaths occur. This misinterpretation has led to preventable deaths and given rise to guideline-associated rabies phobia (GARP). We propose five revisions to refine—not discard—the rule. Reshaping the paradigm is an ethical prerequisite for achieving ‘Zero by 30’.

## Introduction

WHO’s ‘Zero by 30’ strategic plan has accelerated global efforts to end dog-mediated human rabies deaths (1). Post-exposure prophylaxis (PEP) is highly effective when delivered correctly and promptly. Yet a persistent implementation gap is undermining this progress: the 10-day observation rule – a guideline provision designed for ideal, high-resource settings – often fails to achieve its intended outcomes in the real-world contexts where 95% of rabies deaths occur.

The scientific basis of the 10-day observation rule comes from two key studies. Tepsumethanon and colleagues found that naturally infected rabid dogs and cats all die within 10 days of becoming infectious (2, 3). Based on this evidence, WHO recommends that for domestic dogs and cats, if the animal remains healthy during a 10-day observation period after a bite, PEP can be discontinued (4). This policy brief does not dismiss the value of this guideline provision, but rather re-examines its applicability in light of new evidence and real-world implementation challenges, and proposes complementary refinements.

The strict preconditions on which the 10-day observation rule depends are often not met in high-burden settings.

The 10-day observation rule was designed for low-risk, high-resource environments, requiring the biting animal to be owned, confined, have two documented rabies vaccinations, be observable by a veterinarian, and the exposure to occur in a low-risk area (4). However, in high-burden, low-resource settings exemplified by rural China, dog vaccination coverage is only 10–20% (5), most biting animals are free-roaming or stray, and veterinary services are severely lacking. Consequently, the Chinese CDC explicitly advises against using this guideline provision (6), and in 2026 the Anhui CDC stated that exposed individuals should receive standard care promptly (7). This position is consistent with the latest national standard, which does not explicitly endorse the 10-day observation rule and in fact does not mention it at all. Instead, it effectively restricts the rule’s applicability by mandating that Category II exposures on the head and face be managed as Category III when the animal’s health status cannot be confirmed (8). Nevertheless, the provision remains in the WHO guidelines without sufficiently prominent warnings, fostering the dangerous public misconception – “wait 10 days, if the animal is still alive, I do not need vaccination.”

## Special populations: immunocompromised individuals

The 10-day observation rule was not designed for immunocompromised patients. In individuals with impaired immune responses, seroconversion after PEP may be delayed or inadequate, and the provision’s inference of “animal healthy → patient safe” may fail. WHO guidelines explicitly state that immunocompromised individuals should receive full PEP regardless of animal observation (4). This exception shows that the 10-day observation rule does not cover this important population, and in clinical practice the immune status of patients is often not immediately known, further limiting the provision’s general applicability.

## Global evidence of the implementation gap

### China: fatal misconceptions and system fragility

Two publicly reported sentinel events illustrate the real-world fatal consequences of misinterpreting the 10-day observation rule.

In 2020, a 12-year-old girl in Hangzhou was licked on a hangnail by her family’s unvaccinated puppy. She did not seek medical care. Two months later, her younger brother was bitten by the same dog and received rabies vaccination; only then did the girl disclose her own exposure. Because the dog remained healthy and the family had been told that under the “10-day observation rule,” “if the dog does not die within ten days, there is no problem,” they did not take her to a doctor. The girl later died of rabies (9).

In January 2026, a 47-year-old woman in Changsha was lightly bitten on the finger by her own puppy. The wound was tiny and did not bleed. Believing that “a domestic dog is safe” and that “a tiny wound is nothing to worry about,” she did nothing but observe. Two months later she developed rabies and died within days. The Hunan Provincial CDC subsequently clarified that the public frequently misinterprets “observation” as a primary management strategy, rather than an ancillary confirmation tool after standard care has been initiated (10).

These cases highlight a broader systemic vulnerability. An analysis of 240 human rabies cases in Hunan province (2019–2024) showed that the median incubation period for head-and-face exposures was 16 days. The majority of PEP failures involved category III exposures, and weak primary care infrastructure, inadequate wound washing, and delayed presentation were consistently observed (11). These data indicate that in low-resource settings, the very precondition of “immediate, standard PEP” on which the 10-day rule depends is difficult to guarantee. This reality makes lay misinterpretation of the rule particularly dangerous.

These cases underscore that the current presentation of the rule in WHO guidelines is insufficiently safeguarded against misinterpretation. Preventable deaths will continue to occur unless the rule is accompanied by clear, prominent operational safeguards suited to low-resource settings.

### Brazil: misapplication driving resource waste

In São Paulo state (2013–2017), among 572,889 dog and cat bite PEP notifications, the most frequently recommended measure was “observe the animal for 10 days” (44.4%), with a proportion used inappropriately. More than 112,000 individuals who received a correct PEP recommendation did not receive adequate treatment, leading to 247,000 excess vaccine doses and 9,000 excess immunoglobulin doses (12). Misuse of this guideline provision as a substitute for timely PEP drives subsequent panic-driven revaccination and healthcare waste. The economic burden stemming from misapplying the 10-day observation rule is quantifiable; where resources are constrained, every unnecessary vaccine dose diverts funding and manpower away from higher-priority public health initiatives.

### India: cognitive barriers to adherence

A 2025 Indian systematic review reported overall PEP adherence of only 57%, with misunderstanding of the 10-day observation rule identified as a key barrier (13). Patients mistakenly treat the 10-day observation as an alternative to PEP rather than an ancillary confirmation tool after vaccination has been initiated, thereby missing the critical 24–48 h window for prophylaxis.

### Multiple African countries: systemic barriers

Multiple African countries face fundamental precondition deficits in implementing the 10-day observation rule. A 2026 mixed-methods study from Zimbabwe found dog rabies vaccination coverage of only 57.5%, far below the 70% herd immunity threshold; among 14 closely followed dog bite cases, 78.6% involved unvaccinated or status-unknown dogs (14). In the Kilosa district of Tanzania, mass vaccination campaigns reached only 24.4% of the dog population, and 71% of residents were unaware that local dog rabies vaccination campaigns had ever taken place (15). Beyond vaccine coverage and awareness barriers, even the basic act of tracking the biting animal proves operationally challenging: a community-based surveillance project in Malawi followed 610 suspected rabies exposures and found that only 39.2% could be tracked for the full 10-day period. This dataset represents the only community-level field dataset addressing traceability barriers in sub-Saharan Africa and strongly suggests that more than 60% of exposed individuals cannot verify the animal’s outcome, a critical gap now warranting urgent formal validation.^1^ Together, the evidence from these three countries converges on a single conclusion: in high-burden settings, the core preconditions of the 10-day rule are almost never met. The problem is not poor implementation; it is fundamental infeasibility.

### The United Kingdom: a more conservative 15-day window

Even in the UK, a rabies-free country with high veterinary capacity, official guidelines adopt a more conservative 15-day observation period: treatment can be discontinued if a domestic dog or cat remains healthy for 15 days after exposure (16). This “absolute safety” strategy suggests the 10-day standard may have limited safety margins globally. The UK has been free of terrestrial rabies since 1902. Its 15-day window is informed by viral shedding data showing that dogs and cats can excrete virus up to 10 days before clinical signs appear. This means the 10-day observation rule may not capture all potentially infectious periods. The UK’s approach requires owned and traceable animals, reliable veterinary supervision, and high vaccination coverage. These conditions are largely absent where 95% of rabies deaths occur, where most biting animals are free-roaming and vaccination rates fall below 20%. The UK example thus underscores that observation-based protocols are only feasible where their prerequisites already exist.

Biological risks of delayed PEP under misused 10-day observation rule.

From a biological perspective, the misapplication of the 10-day observation rule—specifically the dangerous clinical practice of waiting to initiate or complete full PEP until the 10-day animal observation window concludes—is fundamentally medically indefensible and equivalent to gambling with patient survival, for three critical biological reasons. First, symptomatic rabies carries a nearly 100% case-fatality rate with no effective curative therapy available once neurological signs develop. Second, a single vaccine dose cannot induce neutralizing antibody titers high enough to reliably block viral neuroinvasion and prevent clinical disease. Third, the incubation period after craniofacial Category III bites can be as brief as 5–10 days, a timeline shorter than the full 10-day observation cycle. A systematic review of 122 fatal breakthrough infections further confirmed that 69% of lethal cases involved severe head, face or neck wounds (17). Notably, the official WHO 10-day observation framework mandates immediate initiation of PEP on day 0 alongside animal isolation; the 10-day observation only permits discontinuation of subsequent vaccine doses if the animal remains healthy, and never authorizes delaying the initial prophylaxis course. In China, completing the full PEP course is strongly recommended even if the animal survives the 10-day observation period, as it confers complete immunity, eliminates the need for re-vaccination and passive immunization upon re-exposure, and substantially reduces the financial burden on patients. This practice indirectly reflects the tension between Chinese national guidance and the WHO framework, where the 10-day observation rule remains a general recommendation without explicit risk stratification. For high-risk Category III exposures to unvaccinated stray animals, such delayed PEP practice creates an unacceptably high fatal risk and should never be permitted.

### Another silent epidemic: guideline-associated rabies phobia

Rabies phobia is not uncommon in clinical practice. A 2016 paper described six patients with this condition. They had uncontrollable fear, pronounced anxiety and autonomic symptoms, and repeatedly sought medical care to relieve their distress (18). Yet that report described only the superficial features and did not explore the root cause. Based on many years of front-line clinical experience at our centre, we have found that the difficulties faced by some patients with rabies phobia do not arise from simple personal anxiety. Rather, they are driven by a systemic conflict between WHO guidelines and national public health advice. We therefore propose the concept of guideline-associated rabies phobia (GARP), driven by the conflict between WHO guidelines and national public health advice, and offer the following diagnostic criteria for clinical practice. All three are required:

- A history of animal exposure (bite, scratch, or mucosal lick) during which the patient misinterpreted the 10-day observation rule as a substitute for immediate PEP, and consequently delayed or did not initiate PEP in a timely manner. The patient later came to learn, often by chance, that this interpretation constituted a misuse of the guideline’s original provisions. Persistent anxiety follows this realisation.
- Ongoing anxiety with repeated fixation on the 10-day observation rule and the missed opportunity for timely PEP, even when the animal remained healthy for 10 days or the patient later completed a full PEP course.
- Repeated clinic visits for extra vaccine doses or reassurance beyond standard protocols, driven by an inability to accept that the actual risk is negligible, to the extent that it interferes with normal daily functioning, work, or social interactions. Additionally, patients often engage in compulsive information-seeking about rabies, with each new piece of information paradoxically heightening rather than relieving anxiety, trapping them in a self-reinforcing cycle of fear and reassurance-seeking.

These criteria separate GARP from ordinary rabies anxiety. Common rabies fear is short-lived, resolves after PEP completion, and does not involve prior misuse of the 10-day observation rule or compulsive information-seeking behaviour. GARP, in contrast, arises from the dissonance between acting on a misunderstanding of the rule and later recognising that decision was flawed. Its hallmark is the failure to feel safe even when objective evidence shows minimal risk.

Such patients are not rare in China, nor are they limited to any particular age or educational level. The WHO originally designed the 10-day observation rule as a tool for “scientific reassurance.” In practice, however, the public often overlooks its stringent preconditions. Misled by its name, they treat it as a “wait-and-see” instruction. Patients who act on this misunderstanding spend ten days in acute anxiety, only to find the animal remains healthy. By their reading of the rule, they should now be declared safe. But in China, the rule is officially deemed “not applicable” in most exposure settings, and authorities repeatedly caution that it cannot serve as a management basis. These patients, despite having negligible biological risk, are thus denied the official clearance they expected. They fall into a cycle of repeated vaccination and compulsive reassurance-seeking—suffering psychologically while also consuming public health resources and adding avoidable strain to the system. In severe cases, the condition disrupts daily life, work, or social functioning. Neither verbal reassurance from doctors nor the animal’s survival can break this cycle, because the very rule designed to offer closure has been officially invalidated in China. The transmission risk gradient further exposes the paradox at the heart of the 10-day rule’s misuse. Without timely PEP following exposure to a potentially rabid animal, a single bite to the head or neck carries a mortality probability of 0.535; a scratch to the head or neck, 0.002; a scratch to the upper extremities, 0.001; a lick to broken skin ranges from 0.000001 to 0.0001; and a lick to intact skin bears zero lethal risk (19). Non-bite exposures carry negligible biological hazard, yet they constitute a large share of clinical consultations triggered by misinterpretation of the guideline. This stark mismatch between objective risk and subjective distress fuels the anxiety that defines GARP. More strikingly, a scoping review systematically screened 1,093 dog-bite-related publications, of which 693 reported on sequelae. The great majority focused on the physical harms of rabies (61%), whereas the psychological impacts – anxiety and post-traumatic stress disorder – were rarely addressed and have long been neglected by the research community (20). This silent epidemic should no longer be overlooked. Psychological harm is not an acceptable price to pay for preventing death. We propose the concept of GARP precisely to bring this issue into the scope of WHO guideline revision.

### From evidence to a paradigm crisis: what the 10-day observation rule reveals about guideline development

Cross-regional evidence suggests that the implementation gap of the 10-day observation rule is not a local anomaly but a global systemic challenge. This challenge exposes two deep paradigm defects in WHO’s guideline development:

1. One-size-fits-all thinking: Guidelines are designed for ideal, high-resource environments without offering stratified guidance for countries at different epidemiological, economic, and health-system levels. The core assumption – that implementation conditions are universally adequate – does not hold in reality.

2. Insufficient attention to implementation science: Guideline development largely relies on expert opinion and laboratory evidence from high-income countries, with limited field-testing in low-resource settings (21). The preconditions that the 10-day observation rule requires – owned, traceable animals with high vaccination coverage – are almost never met in high-burden countries, yet the guideline did not anticipate these implementation barriers.

This paradigm defect is not unique to the 10-day observation rule but is a common problem in global guideline development. When guidelines are detached from real-world implementation conditions, even scientifically sound provisions can cause harm.

A call for tiered, implementation-oriented guidelines.

We propose five recommendations to refine – not discard – the 10-day observation rule:

1. Revise the provision with an explicit warning: Downgrade it from a “general recommendation” to a “conditional option under specific circumstances,” and attach an unavoidable red-box warning: “This provision is best restricted to owned, vaccinated, confined animals in low-risk areas. It should not be used as a substitute for immediate PEP. In high-burden settings or when preconditions are not met, immediate full PEP is strongly recommended.”
2. Develop resource-stratified appendices: Provide “must-do” and “should-do” checklists for high-, medium-, and low-resource settings. For low-resource areas, the provision could be omitted entirely, emphasising only “immediate wound washing + full vaccination.”
3. Invest in frontline implementation capacity: Provide tiered training for grassroots providers, develop simple clinical decision aids (flowcharts, mobile-based tools), and establish regional quality control centres.
4. Consider renaming to eliminate ambiguity: The term “observation” implies passive waiting, whereas the provision’s essence is “confirming whether PEP can be terminated early, provided that it has already been initiated.” A change to the “10-day exclusion rule” or “10-day safety confirmation rule” could help convey that it is a post-exposure verification tool, not a substitute for immediate PEP.
5. Consider explicit contraindication for Category III exposures and unvaccinated stray animals: For Category III exposures, particularly craniofacial bites, the biological risk of waiting outweighs any benefit of observation, and the rule should be explicitly contraindicated. For unvaccinated stray or free-roaming animals, the preconditions for safe application of the 10-day rule, including ownership, traceability, reliable veterinary observation, and documented vaccination history, are almost never met. For owned, vaccinated, and reliably observable animals in low-risk Category II exposures, the rule retains value provided PEP is initiated immediately on day 0.

These findings support a differentiated policy response. We do not advocate complete removal of the rule; rather, we argue for conditional application with explicit safeguards.

## Conclusion

The key to achieving ‘Zero by 30’ is not a single perfect rule for all settings, but a flexible, implementation-oriented framework that respects local realities.

## Footnotes

1 Mastala et al. (22): Preprint, not peer-reviewed; cited exclusively as preliminary field observation for contextual discussion, not as definitive population evidence.
